# Supplementary material for: A Gain-of-Function Mutation on BCKDK Gene and Its Possible Pathogenic Role in Branched-Chain Amino Acid Metabolism
Source: Genes (Basel). 2022 Jan 26;13(2):233. doi: 10.3390/genes13020233 (PMC8872256; doi:10.3390/genes13020233)
Supplement: Supplementary file 1 [file genes-13-00233-s001.zip › genes-1543438-supplementary.pdf]

A gain of function mutation on *BCKDK* gene and its possible pathogenic role in branched-chain amino acid metabolism

Supplementary Material

Section SM. Modeling

Due to the lack of 3D-crystallographic structures of the human BCKDK (hBCKDK), we used the structures of the rat BCKDK (rBCKDK) which have already been solved experimentally. In order to use such structures as a basis for our hypotheses on the role of the histidine residue at position 162 in the human species, the sequences of the BCKDKs of the two species were aligned in pairs and the result was 95.6% sequence identity (see **Figure S1).** The two sequences around our residue don't change. Indeed, the residues from 112 to 357 are the same in rat and in human.

Section SMD. Molecular Dynamics simulations

We performed MD simulations for wt and p.H162Q BCKDK using three X-ray structures (see **Table S2**): rBCKDK in complex with (S)-α-ClC (PDB code 3TZ0), α-KIC (PDB code 4H7Q) and without any inhibitor (PDB code 1GKX). Since the intracellular loop (*residues 302 – 336 in 3TZ0 PDB-structure; residues 307 – 334 in 4H7Q PDB-structure; residues 304 – 336 in 1GKX PDB structure*) is not present in the structure, it was modelled using the Swiss-Model webserver[1]. Each was equilibrated through a complete MD simulation workflow: steepest descent minimization, NVT and NPT equilibration, and MD production under the NPT ensemble, see methods section in the main text for more details. To verify whether the systems were well equilibrated the root mean square deviation (RMSD) of BCKDK backbone atoms for each was calculated (**Figure S2**). In presence of the synthetic inhibitor ((S)-α-ClC) (**Figure S2 A)**, the system reaches the equilibrium at 300 ns, whereas in presence of the physiological inhibitor the system reaches the equilibration state at 200 ns (**Figure S2 B)**. Without inhibitor the system is equilibrated at 150 ns for the wt and at 200 ns for the p.His162Gln (**Figure S2 C)**. These differences may be explained by the fact that different X-ray structures were used.

Next, the root mean square fluctuation (RMSF) for each system was calculated in order to observe the regions of flexibility in our protein (see **Figure S3**). It seems that, at position 162, and in presence of the inhibitors, the glutamine residue is more flexible compared to the histidine residue of the wildtype structure (**Figure S3 A**). This may be explained by the fact that the histidine residue makes a H-bond with the carboxylate group of the inhibitors[2]. Instead, from our MD simulations we observed that the glutamine residue not only does not interact with the inhibitor, but also the presence of the inhibitor induces repulsive forces towards the glutamine residue leading to a higher mobility that consequently promotes an H-bond between the glutamine's side chain and the carbonyl oxygen of the backbone of the Asp164

residue (**Figure S4 A and B**). This effect is not observed in the apo state simulations and it is more evidently observed in the simulation of BCKDK complexed with (S)-α-CIC than with α-KIK.

To gain a deeper understanding on the changes of the dynamics of wt and mutated proteins we performed a principal component analysis (PCA) of the MD trajectories using the PRODY python library[3]. This technique allows us to analyze correlated low frequency motions of the protein. From the porcupine representation for the first principal component (projection of the motion along the first eigenvector) (**Figure S5**), it can be observed that there is a change in the correlated movements along the first eigenvector during the MD simulation for wildtype and p.His162Gln. Indeed, α4 helix in wt (**Figure S5 A**) and in p.His162Gln (**Figure S5 B**) BCKDK structures have a different direction of movement. While we observe a concerted movement of helices α4 and α5 for the wt structure, for the p.His162Gln structure such helices show a divergent movement. This leads to a raise of helix α4 (**Figure S6**) which may contribute to the opening of the active site. Moreover, this substantial displacement of helix α4, which is claimed to be communicated with the lipoyl-binding pocket[2], may also be fundamental for the enzyme activity.

```
95.6% identity in 412 residues overlap; Score: 2048.0; Gap frequency: 0.0%

BCKDK_rat      1 MILTSVLGSGPRSGSSLWPLLGSSLSLRVRSTSATDTHHVELARERSKTVTSFYNQSAID
BCKDK_huma     1 MILASVLRSGPGGGLPLRPLLGPALALRARSTSATDTHHVEMARERSKTVTSFYNQSAID
                 *** *** ***  *  * ****  * ** ************ ******************

BCKDK_rat     61 VVAEKPSVRLTPTMMLYSGRSQDGSHLLKSGRYLQQELPVRIAHRIKGFRSLPFIIGCNP
BCKDK_huma    61 AAAEKPSVRLTPTMMLYAGRSQDGSHLLKSARYLQQELPVRIAHRIKGFRCLPFIIGCNP
                   *************** ************ ******************* *********

BCKDK_rat    121 TILHVHELYIRAFQKLTDFPPIKDQADEAQYCQLVRQLLDDHKDVVTLLAEGLRESRKHI
BCKDK_huma   121 TILHVHELYIRAFQKLTDFPPIKDQADEAQYCQLVRQLLDDHKDVVTLLAEGLRESRKHI
                 ************************************************************

BCKDK_rat    181 EDEKLVRYFLDKTLTSRLGIRMLATHHLALHEDKPDFVGIICTRLSPKKIIEKWVDFARR
BCKDK_huma   181 EDEKLVRYFLDKTLTSRLGIRMLATHHLALHEDKPDFVGIICTRLSPKKIIEKWVDFARR
                 ************************************************************

BCKDK_rat    241 LCEHKYGNAPRVRINGHVAARFPFIPMPLDYILPELLKNAMRATMESHLDTPYNVPDVVI
BCKDK_huma   241 LCEHKYGNAPRVRINGHVAARFPFIPMPLDYILPELLKNAMRATMESHLDTPYNVPDVVI
                 ************************************************************

BCKDK_rat    301 TIANNDVDLIIRISDRGGGIAHKDLDRVMDYHFTTAEASTQDPRISPLFGHLDMHSGGQS
BCKDK_huma   301 TIANNDVDLIIRISDRGGGIAHKDLDRVMDYHFTTAEASTQDPRISPLFGHLDMHSGAQS
                 ********************************************************* **

BCKDK_rat    361 GPMHGFGFGLPTSRAYAEYLGGSLQLQSLQGIGTDVYLRLRHIDGREESFRI
BCKDK_huma   361 GPMHGFGFGLPTSRAYAEYLGGSLQLQSLQGIGTDVYLRLRHIDGREESFRI
                 ****************************************************
```

Figure S1. Sequence alignment of rBCKDK and hBCKDK performed with the EXPASY webserver[4], using the FASTA sequences taken from UNIPROT (entry Q00972 and O14874 for rBCKDK and hBCKDK, respectively). Our residue of interest (His162) is coloured in red.

Supplementary Table S1: Urinary organic acids analysis of the patient at diagnosis and follow-up

**Urinary organic acids**	**2-oxo-isocaproic acid**	**2-oxo-3-methylvaleric acid**	**2-oxo-isovaleric acid**
[Reference Range Values]	[<2 mM/M Cr]	[<2 mM/M Cr]	[<15 mM/M Cr]
At diagnosis	9	6	not detected
At 6-month follow-up	not detected	not detected	not detected

Abbreviations: mM/M Cr, millimolar to molar of creatinine

Table S2 - Overview of the simulated systems.

System	Protein Structure (PDB id)	Duration of the MD simulation (ns)
wildtype BCKDK+ (S)-α-CIC	3TZ0	500
p.His162Gln BCKDK + (S)-α-CIC	3TZ0	500
wildtype BCKDK + α-KIC	4H7Q	500
p.His162Gln BCKDK + α-KIC	4H7Q	500
Apo wildtype BCKDK	1GKX	500
Apo p.His162Gln BCKDK	1GKX	500

A

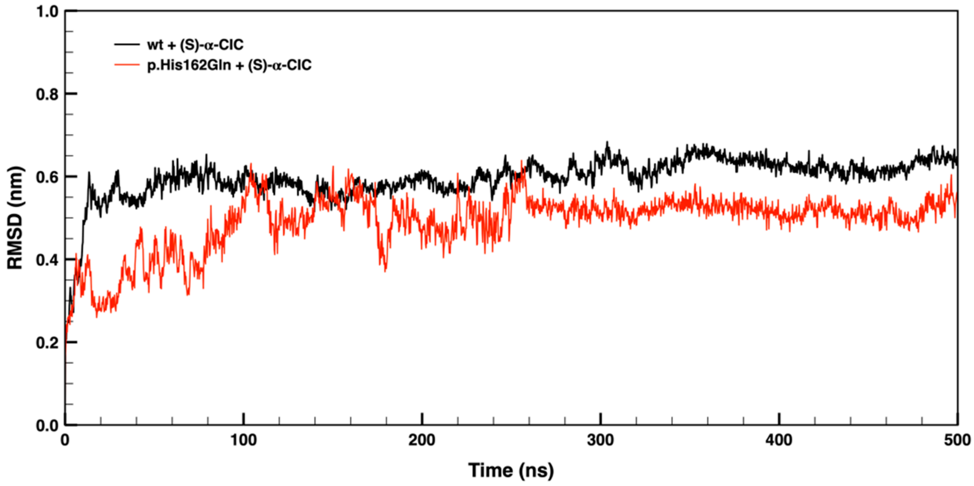

B

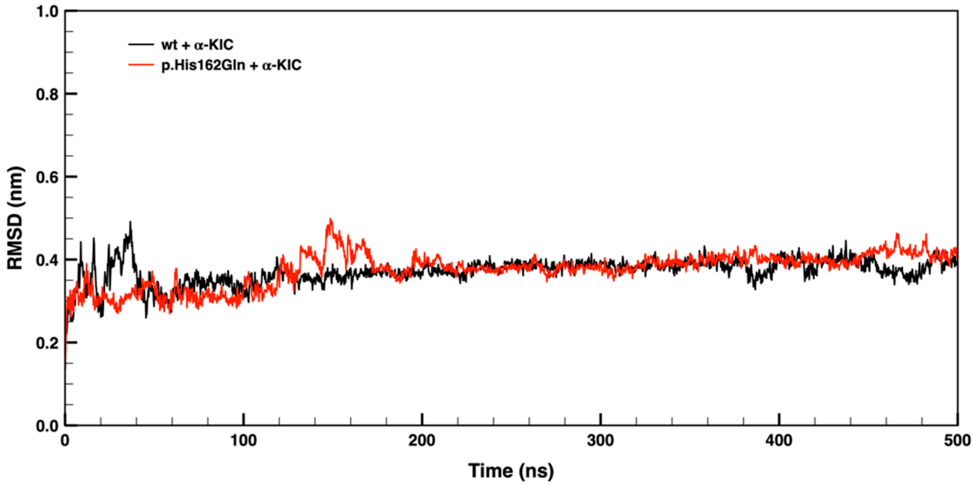

C

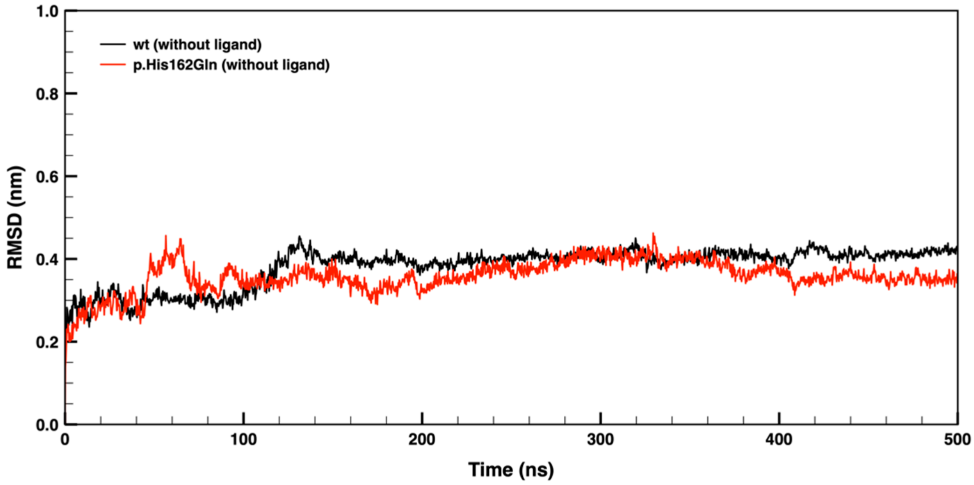

Figure S2. Root mean square deviation (RMSD) of BCKDK backbone atoms of **(A)** wt and p.His162Gln mutant with synthetic inhibitor structures, **(B)** wt and p.His162Gln mutant with α-KIC structures, and **(C)** wt and p.His162Gln mutant structures.

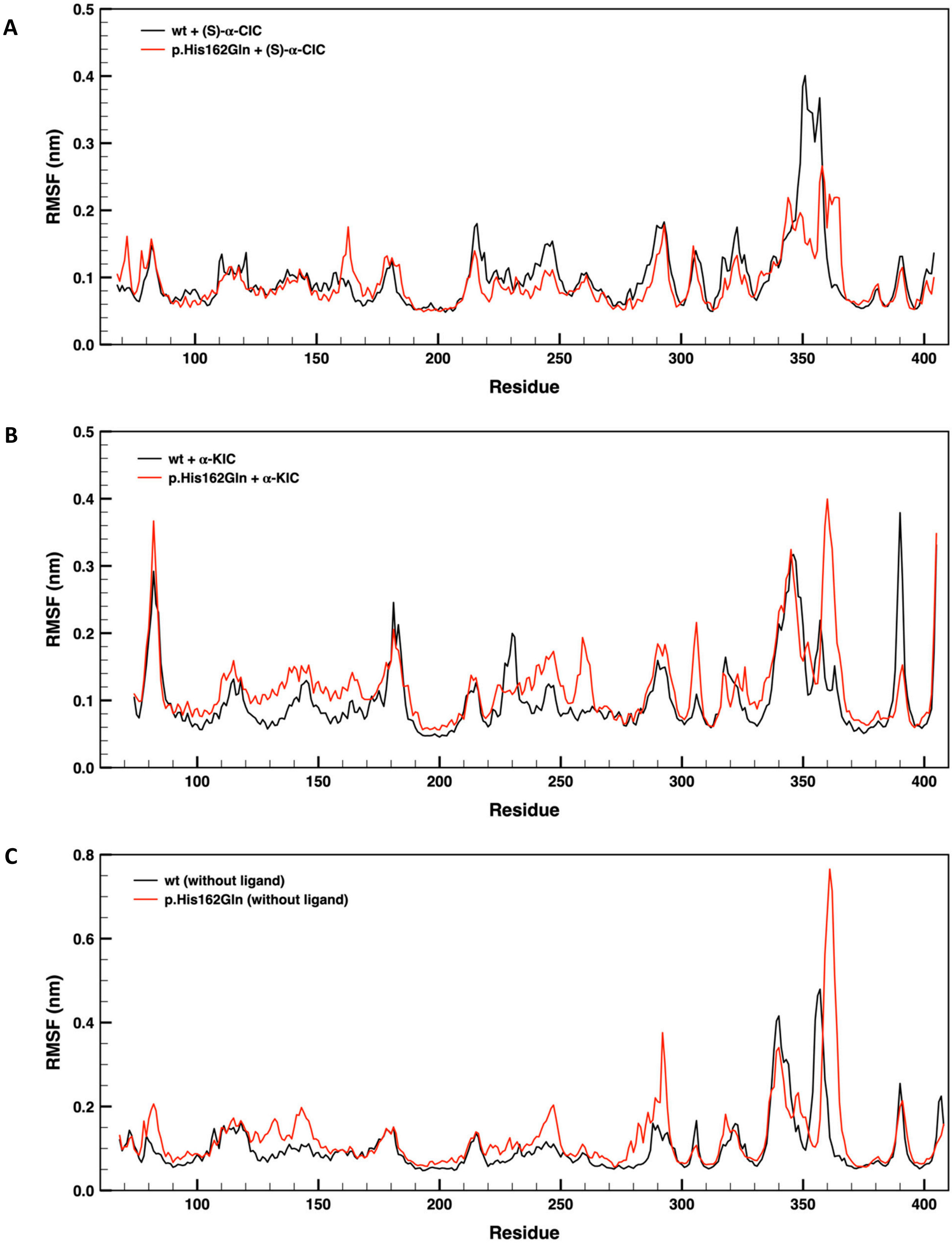

Figure S3. Root mean square fluctuation (RMSF) of BCKDK backbone atoms for (**A**) wt and p.His162Gln mutant with synthetic inhibitor structures, (**B**) wt and p.H162Q mutant with α-KIC structures, and **(C)** wt and p.His162Gln mutant without ligand structures.

A

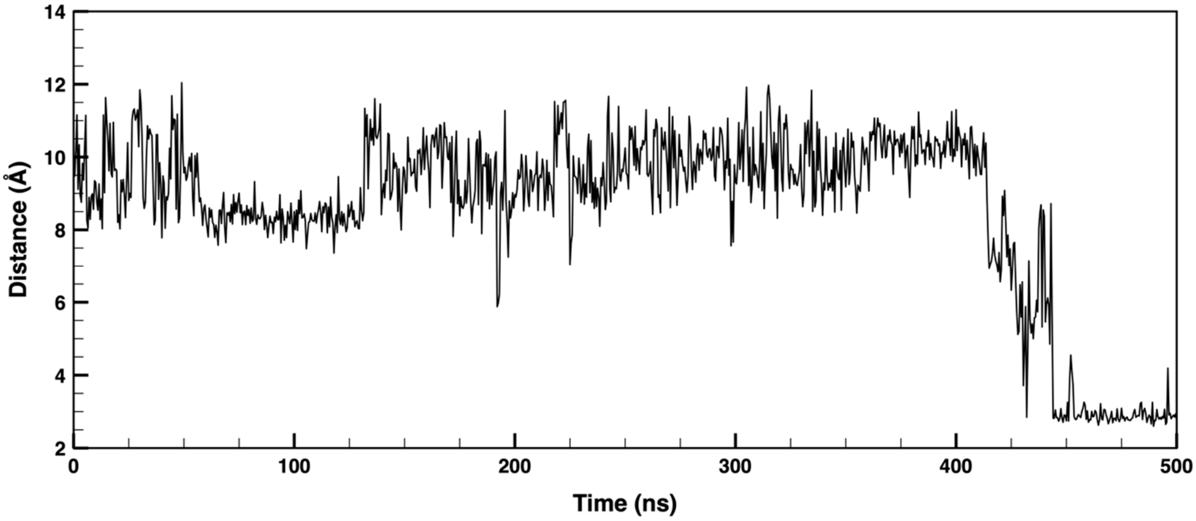

Figure S4. Graphical representation of the distance between the nitrogen of the amino group of the side chain of the Gln162 and the carbonyl oxygen of the backbone of the Asp164 throughout the simulation of BCKDK-(S)-α-ClC complex.

A

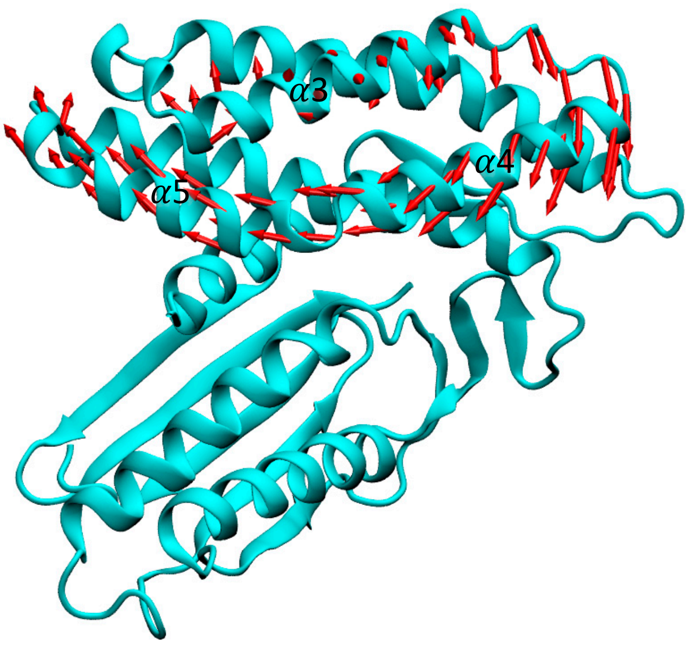

B

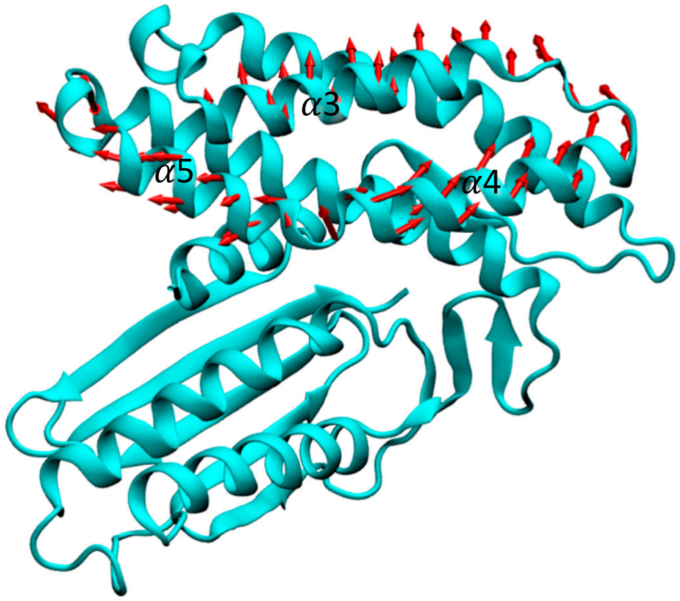

Figure S5. Representation of porcupine plot of the first principal component of the PCA analysis of the MD trajectories for helices $\alpha 3$, $\alpha 4$ and $\alpha 5$ for the **(A)** wildtype structure and **(B)** p.His162Gln structure.

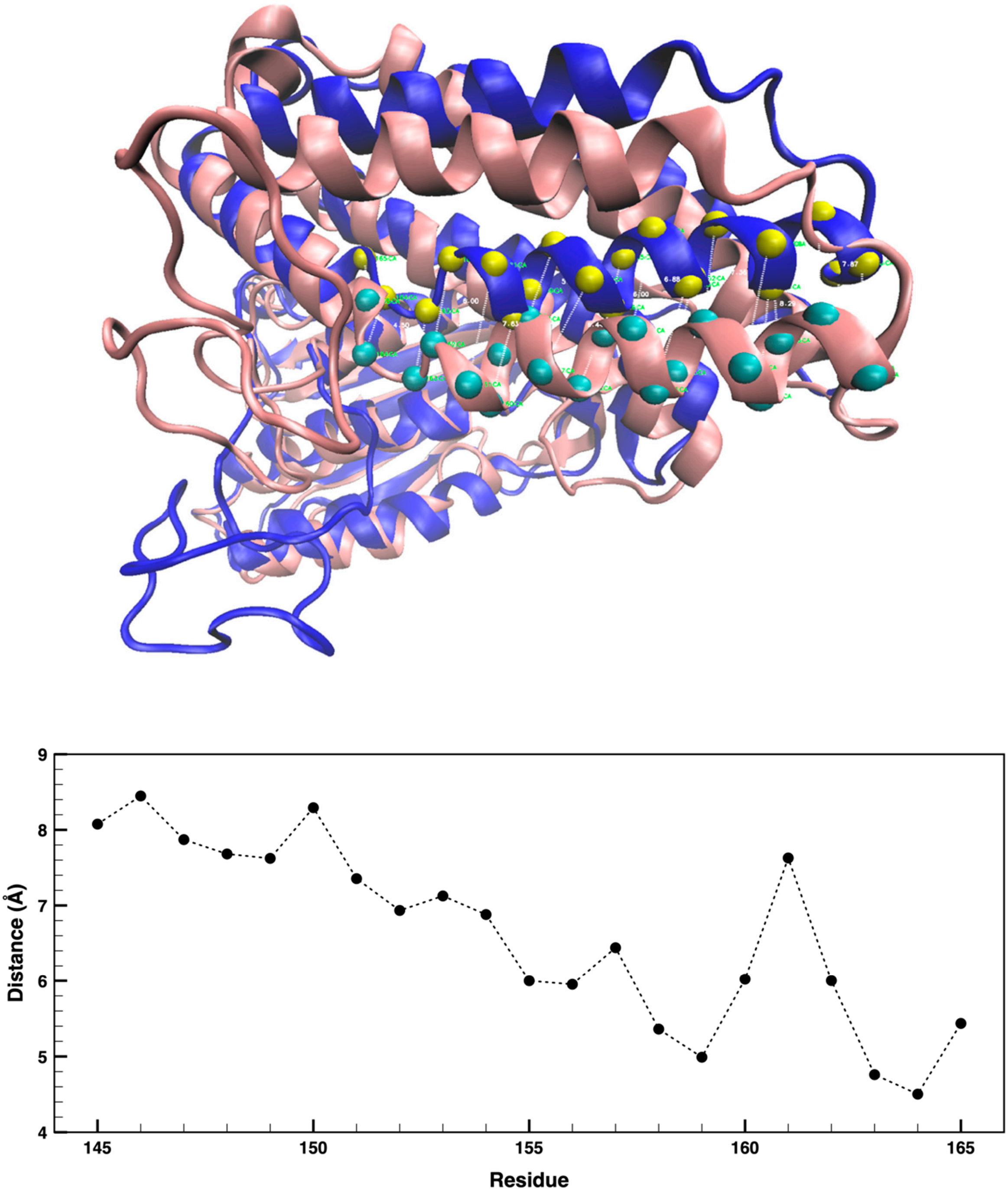

Figure S6. Representation of the distance between the Cα atoms of helix α4 of equilibrated BCKDK wt (pink) and p.His162Gln (blue) structures. The measurements were done after the structural superimposition of representative structures of wt and mutated protein. It shows a significative conformational change at the level of helix α4.

References:

1. Waterhouse, A. *et al.* SWISS-MODEL: homology modelling of protein structures and complexes. *Nucleic Acids Res.* **46**, W296–W303 (2018).

2. Tso, S.-C. *et al.* Structure-based design and mechanisms of allosteric inhibitors for mitochondrial branched-chain α-ketoacid dehydrogenase kinase. *Proc. Natl. Acad. Sci. U. S. A.* **110**, 9728–9733 (2013).

3. Bakan, A., Meireles, L. M. & Bahar, I. ProDy: Protein Dynamics Inferred from Theory and Experiments. *Bioinformatics* **27**, 1575–1577 (2011).

4. Gasteiger, E. ExPASy: the proteomics server for in-depth protein knowledge and analysis. *Nucleic Acids Res.* **31**, 3784–3788 (2003).
